# Enterocin Ent7420 – a potential postbiotic additive: effect on growth, immune response and gut health in MRSE-infected rabbits

**DOI:** 10.3389/fvets.2025.1660371

**Published:** 2025-09-05

**Authors:** Monika Pogány Simonová, Ľubica Chrastinová, Jana Ščerbová, Katarína Tokarčíková, Ľubomíra Grešáková, Rudolf Žitňan, Iveta Plachá, Andrea Lauková

**Affiliations:** ^1^Centre of Biosciences of the Slovak Academy of Sciences, Institute of Animal Physiology, Košice, Slovakia; ^2^Department of Animal Nutrition, National Agricultural and Food Centre, Lužianky, Slovakia; ^3^Department of Animal Morphology, Physiology and Genetics, Faculty of AgriSciences, Mendel University in Brno, Brno, Czechia

**Keywords:** enterocin, postbiotic, methicillin-resistance, staphylococci, health, rabbits

## Abstract

Increasing occurrence of methicillin-resistant (MR) staphylococci in humans and animals leads to special attention because of their difficult treatment and zoonotic character. Therefore, novel natural antimicrobial compounds directed against antibiotic-resistant bacteria are requested to overcome this problem. Currently, bacteriocins/enterocins (proteinaceous substances with antimicrobial activity produced by several lactic acid bacteria/enterococci) present a new promising strategy, both in prevention and treatment. The aim of this work was to evaluate the effect of Enterocin Ent7420 against the MR *Staphylococcus epidermidis* SEP3/Tr2a (MRSE) strain in a rabbit (food animal) model, testing its influence and protective effect on body weight (BW), feed conversion ratio (FCR), phagocytic activity (PA), serum glutathione-peroxidase (GPx) enzyme activity, and jejunal morphology (JM). Ninety-six weaned broiler rabbits were divided into experimental groups S (MRSE strain; to simulate the pathogen attack), E (Ent7420), E + S (Ent7420 + MRSE), and control group (C; without substances). Higher BW and lower FCR (NS) were recorded during Ent7420 application. Decreased JM values (*p* < 0.05) reflect the MRSE strain’s damaging effect on the rabbit organism. Improved parameters of GPx and JM during substance combination suggest that Ent7420 may mitigate staphylococcal pathogenesis, warranting further investigation. These results suggest not only promising preventive use of Ent7420 to improve the growth and immunity of rabbits but also its protective effect against possible staphylococcal (MRSE) infections in rabbit breeding.

## 1 Introduction

The One Health concept integrates optimal human-animal health, sustainable food systems, and environmental protection to address global challenges like antimicrobial resistance (AMR) in the 21st century (1). The incidence of multidrug-resistant infections is on the rise; particularly, pathogenic bacterial species (e.g., staphylococci, enterococci, enterobacteria) capable of ‘hopping’ across these three ecosystems are of interest. From the point of view of AMR, the methicillin resistance and multidrug resistance of staphylococci (MRS, MDRS) have special human and veterinary importance because of their pathogenicity and reservoir of resistance genes. They are also included in a list that categorizes pathogens into critical, high, and medium priority groups and underscores their global impact related to transmissibility, treatability, and prevention options (2); methicillin-resistant *S. aureus* (MRSA) strains maintained their position as high-priority pathogens, posing significant challenges in healthcare settings, mostly in human medicine, causing local pyogenic and systemic infections [toxemia, septicemia; (3, 4)]. While MRSA in humans is detected mainly as hospital- and community-acquired (HA-MRSA, CA-MRSA), it is also capable of transmission between humans and animals as livestock-associated MRSA [LA-MRSA; (5)], causing mastitis, skin lesions, and septicemia in food animals (cows, pigs, rabbits), resulting in economic losses (3, 6). However, in recent years, methicillin-resistant coagulase-negative staphylococci (MR-CNS): *S. epidermidis* (MRSE), *S. haemolyticus*, *S. saprophyticus*, and *S. chromogenes* are of growing concern and have emerged as common causes of various animal diseases, due to their invasiveness, biofilm-forming ability, toxin production, and hemolysins (7). Increasing frequency of MRS and MDRS in animals, mostly in the livestock sector, containing resistance and pathogenicity genes, needs special attention and poses significant challenges for their difficult treatment, zoonotic character, and environmental pollution. All these problems highlight the need to look for new natural antimicrobial substances, e.g., postbiotics, to reduce/treat infections caused by MRS/MDRS of animal origin and prevent AMR transmission, including through the food chain. Postbiotics are functional bioactive compounds produced by food-grade microorganisms, defined as preparations of inanimate microorganisms and/or their components conferring health benefits to the host (8, 9). Postbiotics used for domestic animals contain short-chain fatty acids, polysaccharides, peptidoglycan fragments (muropeptides), organic acids, tryptophan, bacteriocins, enzymes, and cell surface proteins (10, 11). Bacteriocins/enterocins (Ents) constitute a new generation of natural proteinaceous antimicrobials with a broad inhibitory spectrum [produced by several lactic acid bacteria-LAB/enterococci; (12)], as they are non-toxic (13) and do not leave residues except in the case of nisin, when some clinically relevant bacteria express highly specific membrane-associated nisin resistance protein and show resistance to nisin (14, 15). In contrast to nisin, till now, resistance to Ents has not yet been described. Supplementation of bacteriocins/Ents in animal nutrition leads to improvement of growth, immunity, gut health, and productivity of animals and, for their antibacterial activity, are commonly used ATB alternatives (16–19). While the antimicrobial effect of bacteriocins/Ents, especially their anti-MRS activity, has been reported mainly in human medicine and food science (20), the anti-staphylococcal effect of Ents against animal-derived MRS/MDRS has been presented in a few studies, mostly within our team (21–23). Therefore, further studies are needed to monitor and effectively prevent or control MRS occurrence/infections in animals, mainly in food/farm animals/breedings, incorporating these substances into animal feed as beneficial additives to ensure and sustain animal health, productivity, and animal product quality and reduce the antibiotic burden on the environment.

The aim of this study was to evaluate the protective/medicinal effect of Ent7420 against the MR *Staphylococcus epidermidis* SEP3Tr2a (MRSE) strain in a rabbit (food animal) model and its influence on growth performance, glutathione-peroxidase enzyme activity, immune response, and jejunal morphology.

## 2 Materials and methods

### 2.1 Animals and experimental design

The experiment followed a completely randomized design. Ninety-six rabbits (meat lines M91 and P91, weaned at 35 days, both sexes, equal male-to-female ratio per treatment) were stratified by initial body weight and randomly assigned to four treatment groups (24 animals in each) to ensure similar average starting weights across groups. The average live weight of rabbits at the start of the experiment was 1041.3 g ± 132.0. Rabbits were housed in standard cages (61 cm x 34 cm x 33 cm) in a closed building equipped with a heating and forced ventilation system, which allowed the environmental temperature to be adjusted within the range of 20 ± 4°C and relative humidity (70 ± 5%). The photoperiod was 16L:8D. The animals were fed with a commercial pelleted basal diet for growing rabbits (KV, Tekro-Nitra, Ltd., Slovakia; Table 1) with access to feed and water *ad libitum* during the experiment. The ingredients and chemical composition of the diet are presented in Table 1. To determine the crude protein (CP), ash, and dry matter (DM) contents, the Association of Official Analytical Methods (24) protocols were used. Fiber fractions (neutral detergent fiber–NDF includes hemicellulose, cellulose, and lignin; acid detergent fiber–ADF includes cellulose and lignin) were determined using the Van Soest method (25).

**Table 1:** Nutrient content and chemical composition of basal diet on a dry matter (DM) basis.

Nutrient content	g.kg^−1^ in DM
Dry matter	1,000
Crude protein	174.94
Crude fiber	149.29
Crude fat	22.89
Ash	101.6
Starch	269.22
Acid detergent fiber (ADF)	171.08
Neutral detergent fiber (NDF)	332.83
Calcium	17.94
Phosphorus	5.51
Magnesium	2.9
Sodium potasium	1.36
Iron	636.88*
Zinc	110.27*
Copper	23.12*
Total energy value (MJ.kg^−1^)	11.35
Energy-to-protein ratio (kcal/g crude protein)	0.065
Starch to protein ratio (g/g)	1.539

* mg.kg−1 feed, DM- dry matter.

The rabbits in group S (positive control) received the MR *S. epidermidis* SEP3/Tr2a (MRSE) strain [1.0 × 10^5^ CFU/mL; (21)] in their drinking water (through nipple drinkers) at a dose of 500 μL/animal/day for 21 days (from day 1 to day 21), to simulate the spoilage/pathogen attack in rabbits. The strain was marked by rifampicin to differentiate it from the total staphylococci and prepared as described previously by Strompfová et al. (26). Rabbits in group E were administered Ent7420 (produced by the *E. faecium* CCM7420 (27)) at a dose of 50 μL/animal/day, with activity of 25,600 AU/mL in concentration of 0.4 g/L for 21 days. The semi-purified Ent7420 was prepared according to Simonová and Lauková (28). The antimicrobial activity of Ent7420 was determined by the agar spot test (29) against the principal indicator strain *E. avium* EA5 (isolated from a piglet in our laboratory) and expressed in arbitrary units per mL (AU/mL). Animals in the E + S were administered the combination of Ent7420 and the MRSE strain. Control rabbits (group C) had the same conditions, but without additives being applied to their drinking water. From day 21, all animals were fed only the commercial diet. The experiment lasted for 42 days.

### 2.2 Growth performance

Body weight (BW) and feed consumption were measured every week during the experiment; average daily weight gain (ADWG; the difference between the initial and current weight of animals, divided by the number of days that occurred between weights; g/day) and feed conversion ratio (FCR; feed intake divided by weight gain for a period; g/g) were calculated mathematically. Health status and mortality were recorded daily throughout the whole experiment.

### 2.3 Sampling and slaughtering

Blood was sampled from the marginal ear vein (*Vena auricularis*) into dry heparinized Eppendorf tubes at days 0, 21, and 42 for analyses (*n* = 8/group). At days 21 and 42, 8 randomly selected rabbits of approximately similar weights from each group were stunned using electronarcosis (50 Hz, 0.3 A/rabbit/4 s), immediately hung by the hind legs on the processing line, and quickly bled by cutting jugular veins and carotid arteries.

### 2.4 Glutathione-peroxidase and phagocytic activity in blood

The activity of glutathione-peroxidase of blood (GPx; μkat/L) was determined by the colorimetric method (Spectrophotometer UV-2550 Shimadzu, Japan) using the commercial kit Randox RS 504 (Randox Laboratories Ltd., United Kingdom). The direct microscopic counting procedure, using the yeast-cell method, was used for phagocytic activity (PA) analysis in blood (30). Blood smears stained with May-Grünwald and Giemsa-Romanowski stains were used for calculating the number of white cells containing at least three engulfed particles per 100 white cells (monocytes/granulocytes).

### 2.5 Gut morphology investigation

To test jejunal morphology (JM; villus surface area, villus circumference, villus height, crypt depth, and villus height:crypt depth (VH: CD) ratio), samples of proximal jejunum (approximately 4 cm in length) were collected at each sampling day (*n* = 8/group; day 21, 42), flushed with 0.9% saline to remove all the content, and immediately fixed in 4% neutral buffered formalin. After rinsing with water, samples were dehydrated in a graded series of ethanol (30, 50, 70, 90%, and absolute ethanol), cleared with benzene, embedded in paraffin, and sectioned at 5 μm thickness (10 slices of each sample). Sectiones were mounted on glass slides and stained with hematoxylin and eosin. Thirty well-oriented villi per animal were selected for morphometric analysis using a light microscope (Axiolab, Carl Zeiss AG, Jena, Germany) under standardized conditions (31). The histological samples were microphotographed (Nikon LABOPHOT 2 with a camera adapter DS Camera Control Unit DS-U 2), and the NIS-Elements version 3.0 software (Laboratory Imaging, Prague, Czech Republic) was used.

### 2.6 Statistical analysis

All statistical analyses were performed using GraphPad Prism software version 10.5.0 (GraphPad Software, San Diego, California, United States). The study followed a completely randomized design. All data were tested for normality using a Shapiro–Wilk’s test and Brown-Forsythe test to examine homogeneity of variance. The data of growth performance were evaluated using one-way analysis of variance (ANOVA), followed by a Tukey’s post hoc test; data on the JM and GPx activity were evaluated using two-way ANOVA with treatment (4 groups) and time (3 sampling points) as fixed factors. No covariates or random effects were included. Differences between group means were evaluated using Tukey’s post hoc test. Due to the non-normal distribution of PA data, group comparisons were performed using the Kruskal–Wallis’s test, followed by Dunn’s post hoc test for multiple comparisons. Data are presented as mean ± standard error of the mean (SEM), and differences were considered statistically significant at *p* < 0.05.

## 3 Results

The animals were in good health throughout the experiment; no mortality was noted. The BW was affected by both substances (days 0–21; S: by 11.8%; E: by 17.2%; S + E: by 4.3%; Table 2). The highest BW and ADWG were noted during the Ent7420 application (day 21; E vs. C: *p* < 0.01); surprisingly, the lowest ADWG was recorded 3 weeks after its withdrawal (day 42; *p* < 0.001). Increased ADWG was recorded also in groups S and S + E during the whole experiment. The Ent7420 positively affected also the FCR, whereas the lowest FCR (by 4.1%) was recorded compared to C; decreased FCR data were also noted in groups S (by 0.6%) and S + E (by 0.3%).

**Table 2:** The effect of MR *S. epidermidis* SEP3/Tr2a (S), EntA/P (E) and their combinative application (S + E) on growth performance of rabbits.

Parameter	Day	Treatments				SEM^1^	*p*-value^2^
		S	E	S + E	C		
Body weight (g)	0	1,016	1,018	1,053	1,078	26.944	0.3237
	21	1922	1967	1898	1888	48.020	0.6834
	42	2,724	2,690	2,717	2,671	61.813	0.9006
Average daily weight gain	0–21	37.8^ab^	39.5^b^	35.2^ab^	33.8^a^	0.9522	0.0039
(ADWG; g/day/rabbit)	21–42	50.1^a^	45.2^b^	51.2^a^	48.9^a^	0.6888	<0.0001
Feed conversion ratio	0–21	3.13	3.02	3.14	3.15	0.0582	0.3554
(FCR; g/g)	0–42	3.58	3.63	3.73	3.66	0.0551	0.5003

S, MR *S. epidermidis* SEP3/Tr2a; E, Ent7420; S + E, MR *S. epidermidis* SEP3/Tr2a + Ent7420; C, control; ADWG, average daily weight gain; FCR, feed conversion ratio. ^1^Pooled standard error of least square means. ^2^Significance levels of treatment effect. Different lowercase superscripts (a, b) within rows indicate significant differences between treatments at each time point (*p* < 0.05). Statistical analysis was performed using one-way ANOVA followed by a Tukey’s post hoc test.

The treatment effect was observed on tested JM parameters (Table 3). The lowest values of villus circumference and VH: CD were measured after the MRSE strain application, which reflects the negative effect of applied strain on the intestinal epithelium and environment. The opposite results were noted during the Ent7420 application, with the highest villus circumference (day 21; S vs. E: *p* = 0.0203) and VH: CD (day 21; S vs. E: *p* = 0.0208; day 42; S vs. E: *p* = 0.0450); a trend of treatment effect was noted also in the case of villus cut surface area (day 21; S vs. E: *p* = 0.0758). Optimized values measured in S + E show the tendency to improve JM parameters, mostly the VH: CD (day 21; *p* = 0.0620) toward the end of the experiment (almost 3 weeks after the substance withdrawal; day 42). These results reflect the protective effect of Ent7420 against the MRSE infection.

**Table 3:** The effect of MR *S. epidermidis* SEP3/Tr2a (S), EntA/P (E) and their combinative application (S + E) on GPx activity and JM of rabbits.

Parameter	Day	Treatments				SEM^1^	*p*-value^2^		
		S	E	S + E	C		Treatment	Time	Treatment x time
Glutathione-peroxidase (U/Hb)	0	152.0^A^	152.0^A^	152.0^A^	152.0	4.589	0.1614	**<0.0001**	0.0730
	21	227.5^B^	189.5^B^	206.1^B^	190.9	5.601			
	42	192.5^abAB^	215.3^aB^	178.2^abAB^	171.5^b^	6.703			
Villus circumference (μm)	21	1553^a^	1611^b^	1573^ab^	1578^ab^	8.445	**0.0097**	0.4246	0.9863
	42	1,548	1,597	1,564	1,573	7.919			
Villus cut surface area (μm^2^)	21	80,493	83,619	81,351	81,489	494.6	**0.0160**	0.5888	0.9489
	42	79,923	82,841	81,207	81,643	463.6			
VH: CD	21	3.71^a^	4.23^b^	3.79^ab^	3.93^ab^	0.0734	**0.0018**	0.4188	0.9938
	42	3.66^a^	4.12^b^	3.74^ab^	3.86^ab^	0.0692			

S, MR *S. epidermidis* SEP3/Tr2a; E, Ent7420; S + E, MR *S. epidermidis* SEP3/Tr2a + Ent7420; C, control; GPx, glutathione-peroxidase; JM, jejunal morphology; Hb, hemoglobin; VH:CD, villus height:crypt depth ratio. ^1^Pooled standard error of least square means. ^2^Significance levels of main effects of treatment, time, and time x treatment interaction. Different lowercase superscripts (a, b) within rows indicate significant differences between treatments at each time point (*p* < 0.05). Different uppercase superscripts (A, B) within columns indicate significant changes over time within each treatment group (*p* < 0.05). Statistical analysis was performed using 2-way ANOVA followed by Tukey’s post hoc test. The bold values are statistically significant.

The time effect was noted on blood GPx activity; higher values were measured at day 21 in all experimental groups (*p* < 0.0001; Table 3). The treatment effect on GPx was noted only in rabbits receiving Ent7420 alone at day 42, when the highest GPx value was observed (E vs. C: *p* < 0.05; E vs. S, S + E: NS). On the contrary, GPx values decreased in groups treated with the MRSE strain after its withdrawal (S, S + E; day 42).

The Ent7420 application stimulated the non-specific immunity in rabbits, elevating the PA values (Table 4). The highest PA was noted in the S + E group at the end of substances application (day 21; *p* < 0.001), maintaining the immune response of rabbits receiving Ent7420 until the end of the experiment (day 42; E vs. S, C; S + E vs. C: *p* < 0.001).

**Table 4:** The effect of MR *S. epidermidis* SEP3/Tr2a (S), EntA/P (E) and their combinative application (S + E) on PA activity of rabbits.

Parameter	Day	Treatments				SEM^1^	*p*-value^2^
		S	E	S + E	C		
Phagocytic activity	0	57.1				0.4784	-----
	21	60.0^a^	65.0^ab^	72.6^b^	60.1^a^	0.9482	**<0.001**
	42	59.1^a^	79.9^c^	75.1^bc^	60.4^ab^	1.6300	**<0.001**

S, MR *S. epidermidis* SEP3/Tr2a; E, Ent7420; S + E, MR *S. epidermidis* SEP3/Tr2a + Ent7420; C, control; PA, phagocytic activity. ^1^Pooled standard error of least square means. ^2^Significance levels of treatment effect. Different lowercase superscripts (a, b) within rows indicate significant differences between treatments at each time point (*p* < 0.05). Statistical analysis was performed using the Kruskal–Wallis’ test, followed by Dunn’s post hoc test. The bold values are statistically significant.

## 4 Discussion

Despite rabbits’ excellent reproductive and nutritional qualities, weaned juveniles face critical vulnerability to dietary/environmental stresses due to immature immunity, leading to high risks of digestive disorders and infections. The most rapid changes–lack of appetite, stunting, lower gains–are seen in the growth of rabbits. The goal of every breeder is to minimize the negative impact of dietary stress and infections and improve animals´ health and product/meat quality regarding consumers´ safety using natural bioactive substances. Although few studies have evaluated the health benefits of postbiotics in rabbits, current research suggests potential advantages. During the MRSE strain application, no negative impact was noted on the rabbits´ growth, similarly to our previous results recorded during a potentially pathogenic biofilm-forming *Enterococcus hirae* Kr8^+^ strain’s application to rabbits (32). Higher ADWG in rabbits during the Ent7420 application (days 0–21) repeatedly confirms its stimulation effect on growth and nutrient uptake, presented also in our previous studies (33, 34). The growth performance improvement can be explained by several mechanisms/factors, including stimulated cecal metabolism and intestinal enzymes production, enhancement of the host immune system, increased resistance to colonization, and reduced stress in rabbits administering postbiotics (35). Better FCR recorded in the E group also indicates increased feed efficiency due to improved digestibility and nutrient absorption during the Ent7420 application. Improvement of growth parameters were proven also after postbiotic (Culbac®, on the base of stabilized non-viable Lactobacilli fermentation product) treatment of *E. coli*-challenged chickens (36).

Dietary changes and infections often evoke stress in animals, during which the host’s antioxidant system is activated, leading to an immoderate production of reactive oxygen species (ROS) as a reaction to stress (37). GPx enzyme activity in blood is one of the most studied markers of the host organism’s antioxidant system. Elevated GPx values in rabbits receiving the MRSE strain for 21 days prove its possibility to induce oxidative stress in rabbits. Comparing these results to those achieved during a short-term (7 days) application of the MRSE strain (22), we can assume, that the infection period/length could affect the host’s antioxidant response. However, gut microbes can convert redox-active molecules into nutrient sources and electron acceptors to support bacterial growth in the gut. Bacteria, mostly LAB, encode and/or produce antioxidant enzyme glutathione, which directly activates ROS and enhances host antioxidant defenses (38, 39). Bacteriocins can also have antioxidant properties due to their ability to modulate gut microbiota in favor of LAB; higher GPx levels after Ent7420 application correlate with these findings. Hanny et al. (40) also presented the antioxidant activity of plantaricin E and F against enteropathogenic *E. coli* in chickens.

Research on the immunomodulatory activity of bacteriocins is represented by few studies (focusing mostly on nisin), making it difficult to characterize their overall immunomodulatory effects. The immunomodulatory activity of bacteriocins is determined by their structure, concentration and context of their application. Bacteriocins are able to stimulate the innate and adaptive immune system by various mechanisms: maintaining the integrity of the intestinal mucosal barrier, antagonizing pathogens with antimicrobial compounds, and stimulating cytokine and IL-production in macrophages (41, 42). Till now, the *in vivo* immunomodulatory effect of bacteriocins is focused mostly on nisin (42, 43). Modulation of immunity by Ents was described only under *in vitro* conditions (EntAS-48, EntDD14 – decreasing NO production and IL-6 and IL-8 secretion (42)). Increased PA during the Ent7420 application to rabbits demonstrates the stimulation of non-specific immunity; enhanced PA was described during several Ents application in rabbits (44, 45). Phagocytosis by blood leukocytes (monocytes, polymorph nuclear cells) is the host’s defense basic tool against pathogens. Activated phagocytes participate in a mucosal barrier of the intestinal walls, thus inhibiting the transfer of antigens through the intestinal mucosa and supporting the growth of beneficial microbiota. We assume the maintained intestinal barrier during the Ent7420 application; these results point to the ability of Ents to successfully modulate the immune system.

The stable gut environment and homeostasis are formed by a complex of intestinal microbiome, epithelium, and immunity. However, dietary dysbiosis and gastrointestinal infections can alter this stability, disrupting the gut integrity (46). Impaired morphological parameters in rabbits receiving the MRSE strain reflect its negative influence/attack on rabbits´ intestine. In general, beneficial strains and postbiotics/Ents supplemented in the diet are able to strengthen the intestinal barrier, improving the JM parameters, increasing nutrient absorption, and leading also to better health status and higher weight gains in animals (19, 47, 48). Maintaining good digestive health is crucial for nutrient absorption. The highest values of villus circumference, cut surface area, and VH: CD reflect the beneficial effect of the Ent7420, as a larger surface area allows for more contact with digested food and greater nutrient uptake. These results also correlate with higher ADWG and better FCR in rabbits receiving the Ent7420. Improved JM parameters in the S + E group testify to the medicinal effect of Ent7420 applied to MRSE-infected rabbits. Our results with other studies (19, 22) highlight the potential of postbiotics – Ents as dietary supplements to enhance gastrointestinal health in rabbits by strengthening the intestinal epithelial barrier and reducing pathogen translocation.

In conclusion, experimental infection with a potentially pathogenic MRSEP3Tr2a strain in rabbits did not negatively affect the growth and PA but significantly impaired the GPx activity and gut morphology in animals. The Ent7420 application improved weight gains, FCR, and JM and enhanced the immune response and antioxidant defense system in control rabbits (without infection). Ent7420 addition with protective aim in MRSE-infected rabbits mitigate the pathogenic/damage effect of the MRSE strain on rabbits gut health, due to optimized/improved values of tested JM and GPx parameters. Outgoing from the results, the Ent7420 can be used as a potential postbiotic additive in rabbits to maintain health and immunity, improve productivity, and reduce pathogen translocation, offering an effective approach to manage possible infections. Our results also point to the ability of Ents to successfully modulate the immune system, expanding the basic knowledge on the *in vivo* immunomodulatory effect of bacteriocins/Ents. Improvement of intestinal morphology highlights the potential of Ent7420 as a dietary supplement to enhance gastrointestinal health in rabbits. Future studies are needed and should assess Ent7420 in MRSE/MRS infection models while monitoring gut microbiome, cecal enzymatic activity and plasma metabolomes.

## Data Availability

The original contributions presented in the study are included in the article/supplementary material, further inquiries can be directed to the corresponding author.
